# Human umbilical cord mesenchymal stem cell-derived extracellular vesicles ameliorate airway inflammation in a rat model of chronic obstructive pulmonary disease (COPD)

**DOI:** 10.1186/s13287-020-02088-6

**Published:** 2021-01-12

**Authors:** Noridzzaida Ridzuan, Norashikin Zakaria, Darius Widera, Jonathan Sheard, Mitsuru Morimoto, Hirofumi Kiyokawa, Seoparjoo Azmel Mohd Isa, Gurjeet Kaur Chatar Singh, Kong-Yong Then, Ghee-Chien Ooi, Badrul Hisham Yahaya

**Affiliations:** 1 Lung Stem Cell and Gene Therapy Group, Regenerative Medicine Cluster, Advanced Medical and Dental Institute (IPPT), SAINS@BERTAM, Universiti Sains Malaysia, 13200 Bertam, Penang Malaysia; 2 Stem Cell Biology and Regenerative Medicine, School of Pharmacy, University of Reading, Reading, RG6 6AP UK; 3 RIKEN Centre for Developmental Biology, 2-2-3 Minatojima-minamimachi, Chuou-ku, Kobe, 650-0047 Japan; 4 Department of Pathology, School of Medical Sciences, Health Campus, Universiti Sains Malaysia, 16150 Kubang Kerian, Malaysia; 5 Institute for Research in Molecular Medicine (INFORMM), Universiti Sains Malaysia, 11800 Gelugor, Penang Malaysia; 6 CryoCord Sdn Bhd, Bio-X Centre, 63000 Cyberjaya, Selangor Malaysia; 7 USM-RIKEN International Centre for Ageing Science (URICAS), Universiti Sains Malaysia, 11800 Gelugor, Penang Malaysia

**Keywords:** COPD, Umbilical cord mesenchymal stem cells, Extracellular vesicles, An animal model

## Abstract

**Background:**

Chronic obstructive pulmonary disease (COPD) is an incurable and debilitating chronic disease characterized by progressive airflow limitation associated with abnormal levels of tissue inflammation. Therefore, stem cell-based approaches to tackle the condition are currently a focus of regenerative therapies for COPD. Extracellular vesicles (EVs) released by all cell types are crucially involved in paracrine, extracellular communication. Recent advances in the field suggest that stem cell-derived EVs possess a therapeutic potential which is comparable to the cells of their origin.

**Methods:**

In this study, we assessed the potential anti-inflammatory effects of human umbilical cord mesenchymal stem cell (hUC-MSC)-derived EVs in a rat model of COPD. EVs were isolated from hUC-MSCs and characterized by the transmission electron microscope, western blotting, and nanoparticle tracking analysis. As a model of COPD, male Sprague-Dawley rats were exposed to cigarette smoke for up to 12 weeks, followed by transplantation of hUC-MSCs or application of hUC-MSC-derived EVs. Lung tissue was subjected to histological analysis using haematoxylin and eosin staining, Alcian blue-periodic acid-Schiff (AB-PAS) staining, and immunofluorescence staining. Gene expression in the lung tissue was assessed using microarray analysis. Statistical analyses were performed using GraphPad Prism 7 version 7.0 (GraphPad Software, USA). Student’s *t* test was used to compare between 2 groups. Comparison among more than 2 groups was done using one-way analysis of variance (ANOVA). Data presented as median ± standard deviation (SD).

**Results:**

Both transplantation of hUC-MSCs and application of EVs resulted in a reduction of peribronchial and perivascular inflammation, alveolar septal thickening associated with mononuclear inflammation, and a decreased number of goblet cells. Moreover, hUC-MSCs and EVs ameliorated the loss of alveolar septa in the emphysematous lung of COPD rats and reduced the levels of NF-κB subunit p65 in the tissue. Subsequent microarray analysis revealed that both hUC-MSCs and EVs significantly regulate multiple pathways known to be associated with COPD.

**Conclusions:**

In conclusion, we show that hUC-MSC-derived EVs effectively ameliorate by COPD-induced inflammation. Thus, EVs could serve as a new cell-free-based therapy for the treatment of COPD.

**Supplementary Information:**

The online version contains supplementary material available at 10.1186/s13287-020-02088-6.

## Introduction

The pathogenesis of the chronic obstructive pulmonary disease (COPD) is characterized by chronic inflammation that leads to small airway obstruction and emphysema [54]. Systemic analysis for Global Burden of Study 2010 demonstrated COPD to be the third leading cause of death in 2010 [57]. Eighty to 90% of all COPD cases are caused by exposure to cigarette smoke (CS) [14]. Inhalation of CS increases the number of neutrophils, B cells, macrophages, and CD8^+^ T cells in the small airway and lungs. These cells, in turn, release multiple inflammatory cytokines, proteinases, and chemokines that together contribute to the degeneration of lung parenchyma [17, 72]. Symptoms of COPD include chronic cough, dyspnea, and excessive production of sputum while anorexia, fatigue, and weight loss may present in patients with severe COPD [13].

Mesenchymal stem cells (MSCs) are multipotent stem cells capable of differentiating into osteoblasts, adipocytes, and chondroblast lineages [16, 29]. Apart from the bone marrow (BM), MSCs can be isolated from various tissue including the umbilical cord (UC), placenta, adipose tissue (AT), amniotic fluid, and lung tissue [56]. However, UC represents an attractive source of MSCs as UC-MSCs are a less ethical concern like embryonic stem cells, and the isolation of UC-MSCs is non-invasive as compared to BM-MSCs. Besides, UC-MSCs have been shown to have similar efficacy in modulating the inflammation as BM-MSCs [46]. In another study, UC-MSCs depicted a greater proliferation, slower senescence rate, and greater anti-inflammatory effect as compared to BM-MSCs and AT-MSCs, suggesting that UC-MSCs might be a better alternative for stem cell-based therapy [44]. Multiple pre-clinical studies suggest that MSCs have the potential to ameliorate the symptoms of many lung diseases such as pulmonary hypertension, asthma, COPD, and pulmonary fibrosis [23, 35, 52, 93]. In the animal model of smoke-induced pulmonary emphysema, biweekly administration of adipose-derived MSCs decreases the level of inflammation, apoptosis, and alveolar enlargement [70]. The result from the first phase of clinical trials also demonstrates multiple doses of MSCs to be safe when administered in COPD patients while reducing the C-reactive protein at 1 month after transplantation [83]. A study in elastase-induced emphysema demonstrated that two doses of MSCs are better than single-dose MSCs. These effects are by decreasing the TNF-α, neutrophil, and lymphocyte count in bronchoalveolar lavage fluid; the thymus weight; and the severity of hypertension and increased elastic fibre content in the lung [66]. However, a recent study had demonstrated the efficacy of a single dose of UC-MSC in moderate-to-severe COPD patients, where the study was reported the UC-MSC was well tolerated with no clinically significant adverse effects reported, decreased number of exacerbations in COPD Assessment Test (CAT) and mMRC scores, and the patients shown a significantly improved in terms of the quality of life [8].

Recently, an increasing number of researches have focused on studying the therapeutic effects of EVs in various diseases. EVs are small membrane vesicle of multivesicular bodies heterogeneous in size released by a variety of cell types, including MSCs. Extracellular vesicles can be found in body fluids such as milk, saliva, urine, amniotic fluid, and cerebrospinal fluid. There are two commonly studied EVs which are exosomes and microvesicles. Exosomes, the size ranges from 40 to 100 nm, are originated from the inward budding of endosome that forms multivesicular bodies (MVB) and released when the MVB fused with the cell membrane [69], while microvesicles (MV), also known as shed microvesicles with a size ranging from 50 to 1000 nm, are formed by outward budding of the cell membrane [68]. The isolation of EVs can be conducted via various methods, including differential ultracentrifugation, density gradient separation, and immunoaffinity capture [34]. The cargo of EVs is proteins, lipids, messenger ribonucleic acid (mRNA), and microRNA (miRNA) which act as messenger molecules in intercellular communication [58, 91].

Studies have shown that EVs isolated from MSCs mimic the therapeutic effects of MSCs and participate in immunomodulation and regeneration in many animal models; however, MSCs depicted better effect in ameliorating the lung injury as compared to its secreted factors [38, 76]. MSC-EVs have been reported to reduce the infarct size in mouse model of myocardial ischemia/reperfusion injury [50]. MSC-EVs were also capable of alleviating inflammation, oxidative stress, and apoptosis [88]. Besides, the use of EVs has been recently suggested as a potential treatment option for COPD [45, 63]. However, to our knowledge, no attempts have yet been made to compare the impact of MSC transplantation to EV administration for in vivo models of COPD. In this study, we examined the effect of human umbilical cord MSCs (hUC-MSCs) and hUC-MSC-derived EVs on inflammation, airway remodelling, and emphysema in a rat model of COPD. In this study, we opted to use cigarette smoke to induce the inflammation in COPD 2 times/day, 7 days/week for 12 weeks, following the method from Zheng et al. [95] with slight modification. Twelve weeks of cigarette smoke exposure was chosen as our model as inflammation, increased goblet cell count, and emphysema were readily observed in this 12-week model [95].

## Materials and methods

### Preparation of FBS-EV-deprived medium

DMEM/F12 (Thermo Fisher Scientific, USA) supplemented with 10% FBS (Thermo Fisher Scientific, USA) were subjected to ultracentrifugation at 100,000×*g* at 18 h at 4 °C by using the Type 50.2Ti fixed-angle rotor, Optima L-100K Ultracentrifuge (Beckman Coulter, USA). The medium was collected and supplemented with 1% antibiotic antimycotic containing penicillin, streptomycin, and amphotericin B (Thermo Fisher Scientific, USA) and 1% l-glutamine (Thermo Fisher Scientific, USA).

### Cell culture, generation of conditioned media (CM), and isolation of EVs

Human umbilical cord-derived MSC (hUC-MSC) passage 4 was kindly provided by Cryocord Sdn Bhd (https://cryocord.com.my/). Cell preparation was conducted in the Current Good Manufacturing Practice (cGMP)-accredited laboratory. The umbilical cord was shredded and enzymatically digested using collagenase (Worthington Biochem, USA) for approximately 2 h at 37 °C. The mesenchymal cells were isolated from human umbilical cord Wharton’s jelly tissue by passing the tissue through a syringe and needle. hUC-MSCs were cultured in Dulbecco’s modified Eagle’s medium (DMEM)—low glucose (Gibco, USA) supplemented with 10% human serum (Cryocord Sdn Bhd), 100 U/ml penicillin, 100 μg/ml streptomycin, and 0.25 μg/ml amphotericin (Gibco, USA). The hUC-MSCs were cryopreserved using standard cryopreservation protocol until being used in the following research experiment.

hUC-MSCs were characterized using flow cytometric analysis and multilineage differentiation capacity, according to the International Society for Cellular Therapy (ISCT) criteria for MSCs [85]. Positive cell surface markers CD90, CD105, CD73, CD166, and HLA-ABC and negative for haematopoietic markers of CD34, CD45, and HLA-DR were characterized using flow cytometry analysis. Meanwhile, multilineage differentiation adipogenesis, osteogenesis, and chondrogenesis were conducted using commercially available differentiation kit.

hUC-MSC-CM were obtained from hUC-MSC passage 5 to passage 7. The hUC-MSCs were cultured from a density of 4000 cells/cm^2^ in complete medium, made up of DMEM/F12 (Thermo Fisher Scientific, USA) supplemented with 10% FBS (Thermo Fisher Scientific, USA); 1% antibiotic antimycotic containing penicillin, streptomycin, and amphotericin B (Thermo Fisher Scientific, USA); 1% l-glutamine (Thermo Fisher Scientific, USA); and 20 ng/ml basic fibroblast growth factor (bFGF) (Thermo Fisher Scientific, USA) and incubated at 37 °C, in humidified air with 5% CO_2_. After 48 h of culture, the media were changed to FBS-EV-deprived complete medium for the generation of hUC-MSC conditioned media (hUC-MSC-CM). After 72 h, hUC-MSC-CM was collected and concentrated using Amicon® Ultra-15 Centrifugal Filter Devices (Merck Millipore, USA).

For the generation and isolation of hUC-MSC-EVs, hUC-MSCs were similarly cultured as described above. After 48 h, the media were changed to FBS-EV-deprived complete medium. After 72 h, hUC-MSC-CM was collected and subjected to differential centrifugation. First, centrifugation of hUC-MSC-CM was conducted by using Kubota 2420 Compact Tabletop Centrifuge (Kubota, Japan) at 300×*g* for 10 min to remove the dead cells. The supernatant was collected and centrifuged again by using Allegra X-15R Centrifuge Ultracentrifuge (Beckman Coulter, USA) at 10,000×*g* for 30 min to remove the debris, followed by ultracentrifugation at 100,000×*g* for 2 h to precipitate the hUC-MSC-EVs by using the Type 50.2Ti fixed-angle rotor, Optima L-100K Ultracentrifuge (Beckman Coulter, USA). The supernatant was discarded, and the hUC-MSC-EV pellet was washed by resuspending in 1xPBS then re-pelleted by ultracentrifugation for 1 h. The hUC-MSC-EV pellet was collected and resuspended in 150 μl 1xPBS and used fresh for the treatments.

### Transmission electron microscope

Freshly isolated hUC-MSC-EVs in 150 μl of 1xPBS suspension were loaded onto carbon-coated copper grids (Ted Pella, USA) and incubated for 10 min. The grid was blotted with filter paper and stained with 2% uranyl acetate (Ted Pella, USA) for 1 min. Excessive uranyl acetate was removed, and the grid was let dry for 15 min before viewing using Energy Filter TEM Libra-120 (Carl Zeiss AG, Germany).

### Nanoparticle tracking analysis

The particle size of hUC-MSC-EVs was characterized by nanoparticle tracking analysis (NTA) using a NanoSight NS300 (Malvern analytical, UK) blue laser system. hUC-MSC-EVs were diluted with 1xPBS between 1:10 and 1:20 and loaded into the laser module sample chamber. The system focuses the laser beam allowing observing and measuring small particles. Five readings were recorded for each hUC-MSC-EV sample.

### Western blot

β-Actin and CD63 expression were confirmed with western blot analysis; 2 mg/ml of hUC-MSC-EVs was separated by using 12% SDS-polyacrylamide gel electrophoresis (PAGE) and then transferred onto the polyvinylidene difluoride (PVDF) membrane (Bio-Rad). The membrane was blocked with 2% BSA for 1 h at room temperature and incubated with primary antibodies, rabbit monoclonal antibody CD63 (Abcam, Cat. No. ab134045) 1:2000 dilution, and rabbit monoclonal antibody β-actin (Cell Signaling Technologies, Cat. No. 4970S) 1:5000 dilution overnight at 4 °C. The membrane was then washed with PBST and incubated with fluorescence secondary antibody goat polyclonal anti-rabbit IgG (Thermo Fisher Scientific, Cat. No. A16097) 1:10,000 dilution for 1 h at room temperature. The secondary antibodies were washed with PBST and developed using a fluorescence detection system (Licor).

### Animal model of COPD

Male Sprague-Dawley (SD) rats (250–350 g) aged 8–9 weeks (*n* = 36) were obtained from the Animal Research and Service Centre (ARASC), Universiti Sains Malaysia. All animal procedures were approved and performed according to the ethical standards of the Animal Ethics Committee of the Universiti Sains Malaysia [No. USM/Animal Ethics Approval/2016/(104)(812)]. The approved protocols for the animal study were based on the Guidelines for the Care and Use of Animals for Scientific Purposes (USM [43]) which was developed based on the Malaysian Animal Welfare Act (2015) and guidelines by the Australian Codes for the Care and Use of Animals for Scientific Purposes (8th Edition, 2013) and the Singapore Guidelines on the Care and Use of Animals for Scientific Purposes.

**Fig. 1:**
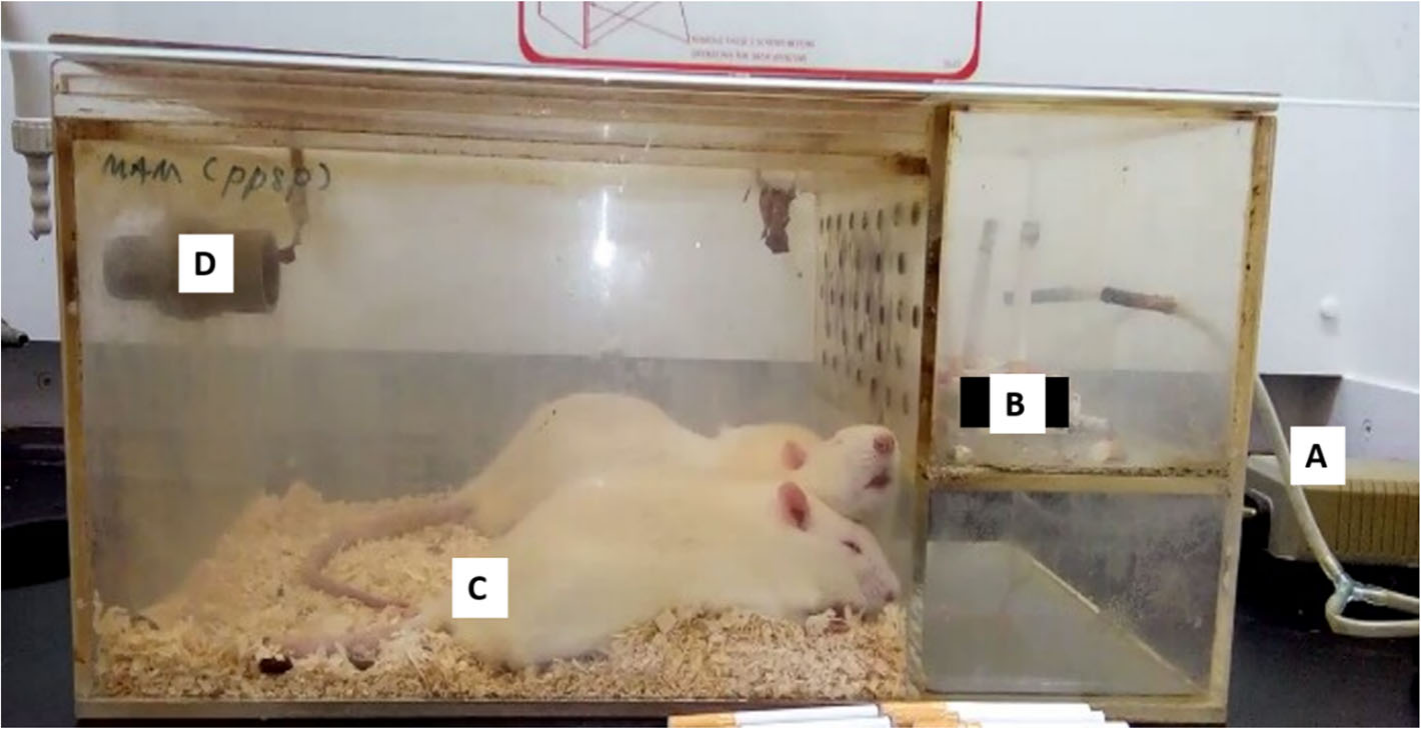
The smoking chamber. For each cigarette smoke exposure session, 3 cigarettes were burnt in the compartment (B) and the smoke produced was continuously ventilated by 2 air pumps (A) to another compartment where the rats were placed (C). Each session lasted for 15 min, and the smoke was simultaneously ventilated out from the chamber into the air through a polyvinyl chloride tube (D)

The in vivo study was conducted in a Good Laboratory Practice (GLP)-accredited laboratory in Animal Research Facilities, Advanced Medical and Dental Institute (IPPT), Universiti Sains Malaysia. The experimental procedure was conducted as previously described by Zheng et al. [95] with slight modifications. COPD symptoms and inflammation were established by using commercially available cigarettes, Marlboro (Philip Morris, USA) (each containing 10.0 mg of tar and 1.0 mg of nicotine). In total, 36 rats were divided into 6 groups (*n* = 6): naive (untreated group), CS (injury group), CSSH (2-week self-healing group), hUC-MSC-EVs (hUC-MSC-EVs-treated group), hUC-MSCs (hUC-MSC-treated group), and hUC-MSC-CM (hUC-MSC-conditioned media-treated group). All groups except naive were exposed to sidestream cigarette smoke for 15 min per session, 6 cigarettes for 2 sessions, 7 days a week, for 12 weeks in a smoking chamber (Fig. 1). Rats were left to rest for 2 h between each session.

Treatments were given via intratracheal delivery in 150-μl vehicle (1xPBS) on day 85 post-cigarette induction. Rats were anaesthetized intravenously by using ketamine (50 mg/kg) and xylazine (5 mg/kg). hUC-MSCs (2.5 × 10^6^), hUC-MSC-EVs isolated from 2.5 × 10^6^ hUC-MSCs, and hUC-MSC-CM concentrated from 2.5 × 10^6^ hUC-MSCs were used in the experiment. The naive and CS groups were euthanized on day 85; meanwhile, the rest of the groups were euthanized on day 99. Rats were euthanized by using intravenous injection of pentobarbital (200 mg/ml) (Dolethal, Lure Cedex, France).

### Peripheral blood collection

Peripheral blood (300 μl) was collected from the rat tail vein and placed into a 1-ml EDTA tube (Greiner Bio-One, Austria) and subjected to whole blood count using the Cell Dyn Hematology Analyzer (Abbott, USA).

### Histological assessment

Haematoxylin and eosin (H&E) staining was performed for the analysis and scoring of peribronchial and perivascular inflammation, alveolar inflammation, and emphysema. Meanwhile, Alcian blue-periodic acid-Schiff (AB-PAS) staining was performed for the analysis of goblet cell count. Scoring of inflammation within the airway was conducted using a semi-quantitative analysis. Slides were blindly coded before a pathologist scored the tissues. The inflammation scoring was performed using the scale of 0 to 3 based on the presence and intensity of inflammatory cell infiltration in the peribronchial and perivascular areas. Two slides were analysed per animal, with a total of 5 animals per group. The score was done according to the following parameters: 0, no inflammation detected; 1, occasional cuffing with inflammatory cells; 2, most bronchi and vessels are surrounded by a thin layer of inflammatory cells (1–5 cells thick); and 3, most bronchi and vessels are surrounded by a thick layer of inflammatory cells (> 5 cells thick). Alveolar inflammation scoring was done by grid on tissue section photos captured by fluorescence microscopy (Olympus, Japan). One hundred points were counted on random areas on the slides. Ten areas were analysed on 2 slides per animal with a total of 5 animals per group. Goblet cells were counted using light microscopy (Olympus, Japan). Five hundred cells were counted, and the number of goblet cells was divided by the total cells to get a percentage of goblet cells. One slide per animal with a total of 5 animals per group was assessed. Emphysema was evaluated by using the mean linear intercept (*Lm*), which measures the enlargement of the alveolar space. Measurement was done by using × 40 objective and × 10 eyepiece, and photos of the sections were taken and superimposed with 30 × 30-μm grid. Ten pictures of 2 slides per animal with a total of 5 animals per group were captured. The number of alveolar intercepts along the gridline was counted and calculated based on the following formula as described previously (Andersen et al, [3]):

$$ Lm=\frac{NL}{m} $$

where:

*N* = number of lines across the photographed area

*L* = length of the line across the photographed area

*m* = number of intercepts

### RNA extraction and microarray analysis

RNA extraction was performed on 30 mg of rat lung from the naive, CS, hUC-MSCs, and hUC-MSC-EVs groups using the RNeasy Mini Kit (Qiagen, Germany) following the manufacturer’s instructions. The purity and concentration of RNA were measured by NanoDrop ND1000 (Thermo Fisher Scientific, US). RNA integrity was determined by Agilent RNA 6000 Nanokit (Agilent Technology, US). cDNA was synthesized and hybridized at 65 °C for 17 h and viewed using the Agilent SureScan Microarray Scanner (Agilent Technology, USA). Comparison between different sample datasets was normalized and analysed using the Gene Spring software. The sample datasets were subjected to *t* test to identify significant changes (*p* < 0.05) between the sample and control groups. Genes with *p* < 0.05 and fold change > 2.0 were filtered as significantly regulated. Volcano plot, heat map, principal component analysis, Venn diagram, and pathway analysis were generated using the Gene Spring software. Gene Ontology analysis using Panther (www.pantherdb.org) was used to classify differentially expressed genes (DEG) by its functional role. GO terms with *p* < 0.05 was considered significantly enriched by DEG.

### Immunofluorescent staining

Immunofluorescent staining was performed to study the expression of NF-κB subunit p65. Briefly, tissue sections were deparaffinized in xylene and rehydrated in graded ethanols. The tissues were blocked with 5% goat serum for 30 min and incubated with primary antibody mouse monoclonal NF-κB-P65 (F-6) (Santa Cruz Biotechnology, USA) 1:200 for 1.5 h in room temperature. After washing with PBS, slides were incubated with secondary antibody Alexa Fluor 555 goat anti-mouse IgG (H+L) (Thermo Fisher Scientific, USA) and counterstained with DAPI 1:2000 in 1xPBS, and viewed under the IX71 Fluorescence Microscope (Olympus, Japan).

### Statistical analysis

Statistical analyses were performed using GraphPad Prism 7 version 7.0 (GraphPad Software, USA). Comparison among more than 2 groups was done using one-way analysis of variance (ANOVA) with Tukey’s multiple comparison test. Data presented as mean ± standard deviation (SD). Differences are considered to be statistically significant when *p* ≤ 0.05, whereas *p* ≤ 0.001 was considered to be highly significant.

## Results

### Characterization of hUC-MSCs

**Fig. 2:**
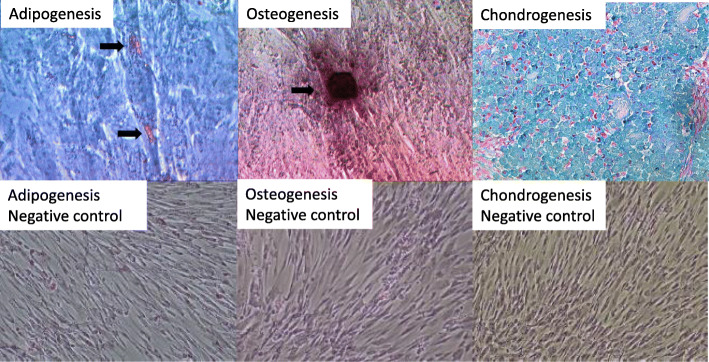
Differentiation of hUC-MSCs. hUC-MSCs differentiate into adipogenesis, osteogenesis, and chondrogenesis, under the differentiation medium. Adipogenesis was evidenced by the formation of lipid droplet stained red, the formation of osteogenesis was evidenced by calcification stained red, and the formation of cell-matrix stained blue evidenced chondrogenesis

Mesenchymal stem cells that were isolated from the human umbilical cord blood were subjected to flowcytometry and differentiation analysis. hUC-MSCs were positive for CD73, CD90, CD105, and CD166 and negative for CD34, CD45, CD31, and HLA DR DP DQ (Table 1). Differentiation analysis showed the ability of MSCs to differentiate into adipocyte evidenced by lipid droplet formation, osteocyte evidenced by calcification formation, and chondrocyte evidenced by cell-matrix formation (Fig. 2).

**Table 1:** The expression analysis of hUC-MSC surface marker using flowcytometry

Surface marker	Expression (%)
CD73	92.4
CD90	93.1
CD105	84.1
CD45	0.2
CD34	0.0
CD31	0.2
CD166	63.1
HLA-ABC	60.9
HLA DR DP DQ	0.0

### Characterization of hUC-MSC-EVs

**Fig. 3:**
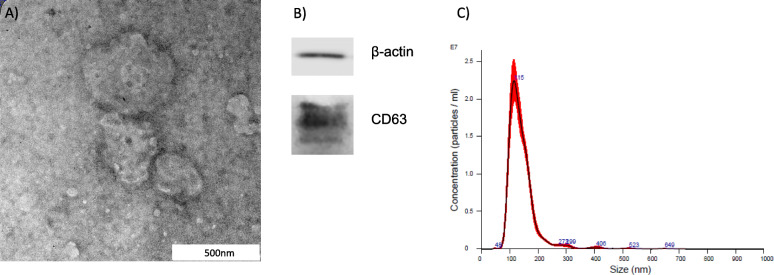
Characterization of hUC-MSC-EVs. **a** Morphological observation using EFTEM of hUC-MSC-EVs showed to be rounded in shape. **b** CD63 expression was observed by western blot analysis. β-Actin is visible at 42 kDa, and CD63 is visible at 30–65 kDa. **c** Particle distribution by Nanosight NS300 reported an average hUC-MSC-EVs diameter of 153 nm. Representative data from three independent experiments

hUC-MSC-EVs were isolated by differential centrifugation to remove cell debris and apoptotic bodies. hUC-MSC-EVs pellet suspended in 1xPBS was characterized based on morphology, size distribution, and protein marker expression. Energy-filtered transmission electron microscopy examination showed hUC-MSC-EVs were rounded in shape with an average size of 200 nm (Fig. 3a). Western blot analysis revealed the presence of the specific exosome marker CD63 at 30–65 kDa and β-actin at 42 kDa (Fig. 3b). Nanoparticle tracking analysis of hUC-MSC-EVs showed an average diameter of 153 nm (Fig. 3c). Table 2 shows the mean, mode, SD, and range of three hUC-MSC-EVs samples used in NTA.

**Table 2:** Analysis of hUC-MSC-EVs size distribution

Sample	Mean (nm)	Mode (nm)	SD (nm)	Range (nm)
1	141.2	115.0	51.4	36–737
2	156.5	116.9	68.4	64–795
3	163.0	123.1	68.3	25–740

### hUC-MSC-EVs decreased lymphocyte count in the peripheral blood

**Fig. 4:**
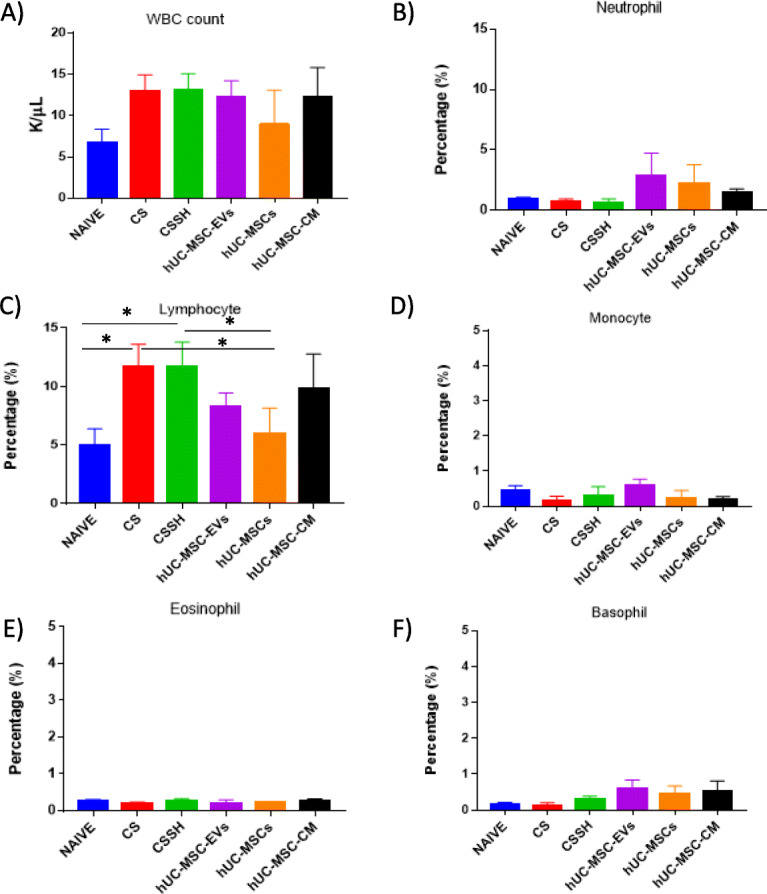
White blood and differential cell counts of the peripheral blood in the naive and injury groups. **a** White blood cell counts of the peripheral blood in the naive and CS groups show an increase in response to CS with no significant decrease following treatments. The following graphs show the differential cell counts for **b** neutrophils, **c** lymphocytes, **d** monocytes, **e** eosinophils, and **f** basophils in the peripheral blood. No significant increase in neutrophils, monocytes, eosinophils, and basophils was observed in CS. However, the percentage of lymphocytes significantly increased in response to CS, followed by a decrease following treatment with hUC-MSC-EVs and hUC-MSCs (*p < 0.05)

To study the effect of hUC-MSC-EVs on the circulating immune cells, the peripheral blood was collected and subjected to full blood count. Figure 4a depicted the white blood cell counts of the peripheral blood. The following graphs show the differential cell counts of (b) neutrophils, (c) lymphocytes, (d) monocytes, (e) eosinophils, and (d) basophils in the peripheral blood. CS exposure for 12 weeks observed a non-significant increase in white blood cell (WBC) count with no reduction seen following a 2-week self-healing rest period without exposure to CS (CSSH). Treatment with hUC-MSC-EVs and hUC-MSC-CM did not reduce WBC counts; however, a non-significant decrease was seen in response to hUC-MSCs (Fig. 4a). Notably, CS significantly increased the percentage of lymphocytes compared to the naive group with no observed mitigation following 2 weeks of self-healing (CSSH). A significant decrease in the percentage of lymphocytes was seen in response to treatment with whole-cell hUC-MSCs (*p < 0.05), whereas a slight non-significant decrease in response to hUC-MSC-EVs and hUC-MSC-CM Fig. 4c.

### hUC-MSC-EVs alleviates airway inflammation

**Fig. 5:**
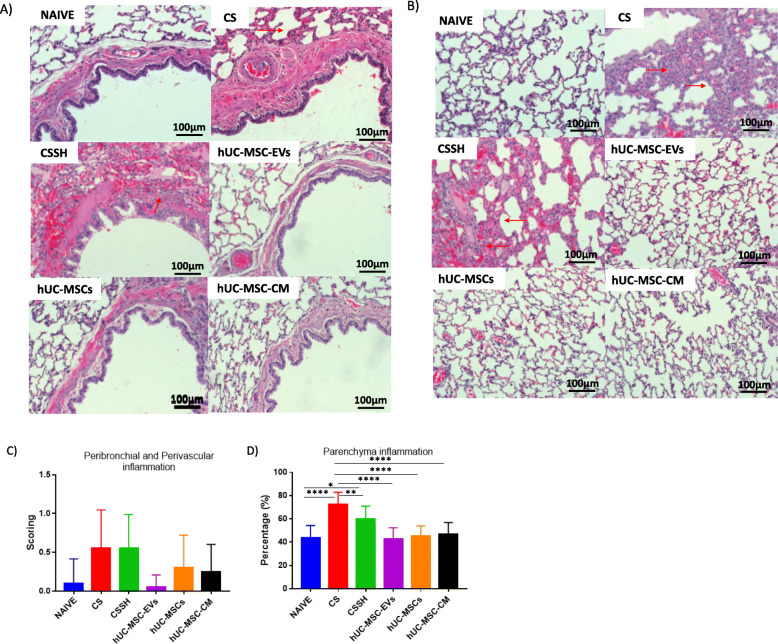
Airway and parenchyma inflammation in the injury and treatment groups. Histological image of peribronchial (**a**). The histological image of parenchyma (**b**). Semi-quantitative histological scoring and analysis of airway inflammation (**c**). Lung parenchymal inflammation (**d**). The arrow on **a** showed accumulation of immune cells in the peribronchial area when exposed to CS for 12 weeks, and self-healing for 2 weeks did not reduce the inflammation. The arrow on **b** showed accumulation of immune cells in the parenchyma area, while 2 weeks of self-healing (CSSH group) did not reduce the inflammation. The scores for inflammation in the airway and alveolar area significantly reduced following treatment with UCMSC-EVs, hUC-MSCs, and hUC-MSC-CM (****p < 0.0001) compared to the CS group

The analysis on histological scoring was conducted on the CS effects on the inflammation in rat airway and lung parenchyma. Figure 5a showed the histological image of peribronchial, (b) histological image of the parenchyma, (c) semi-quantitative histological scoring and analysis of airway inflammation, (d) semi-quantitative histological scoring of lung parenchymal inflammation. The results showed an increase in inflammation scores in response to CS (Fig. 5a, b). The accumulation of immune cells significantly increased in the lung parenchyma. Meanwhile, 2 weeks of self-healing (CSSH) did not reduce the inflammation. However, there was a significant reduction of inflammation scores observed in the parenchyma following treatment with hUC-MSC-EVs, whole-cell hUC-MSCs, and hUC-MSC-CM (**p < 0.001, ***p < 0.0001) (Fig. 5c, d).

### hUC-MSC-EVs reduce the infiltration of the immune cells in the lung

**Fig. 6:**
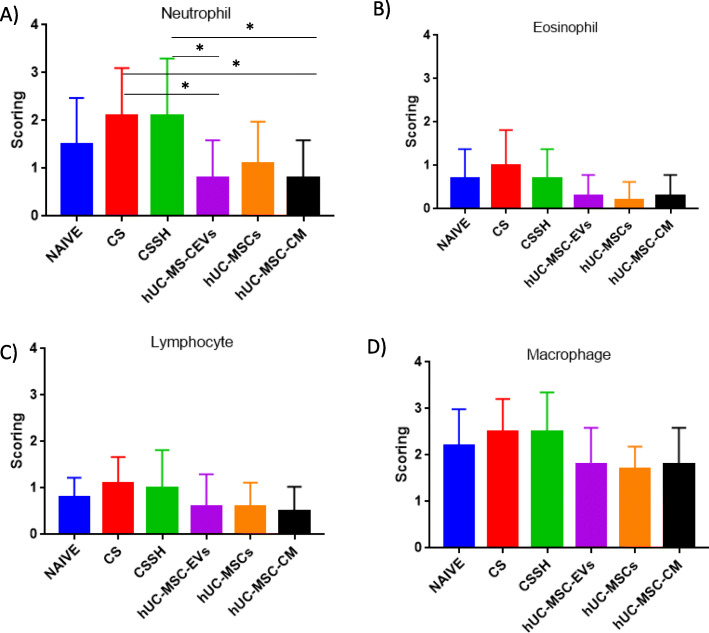
Infiltration of immune cells in rat lung. Semi-quantitative histological scoring and analysis of **a** neutrophils, **b** eosinophils, **c** lymphocytes, and **d** macrophages in the lung. CS increased the infiltration of neutrophils, eosinophils, lymphocytes, and macrophages into the lung. Two weeks of the self-healing period (CSSH) failed to reduce the infiltration of all cells examined. Treatment with hUC-MSC-EVs and hUC-MSC-CM significantly (*p < 0.05) reduced the infiltration of neutrophils

The accumulation of immune cells (neutrophils, eosinophils, lymphocytes, and macrophages) in the lung is a key marker for the development of chronic inflammation in COPD. Figure 6 showed semi-quantitative histological scoring and analysis of (a) neutrophils, (b) eosinophils, (c) lymphocytes, and (d) macrophages in the lung. Our result showed that CS caused an influx of these immune cells into the lung (Fig. 5), predominantly neutrophils and macrophages, while lymphocytes and eosinophils remained present at low levels. Two weeks of self-healing (CSSH) failed to reduce the infiltration of all cell types examined. Notably, the administration of hUC-MSC-EVs, hUC-MSCs, and hUC-MSC-CM significantly reduced the immune cell influx as compared to the CS group.

### hUC-MSC-EVs, hUC-MSCs, and hUC-MSC-CM decreased mucus production

**Fig. 7:**
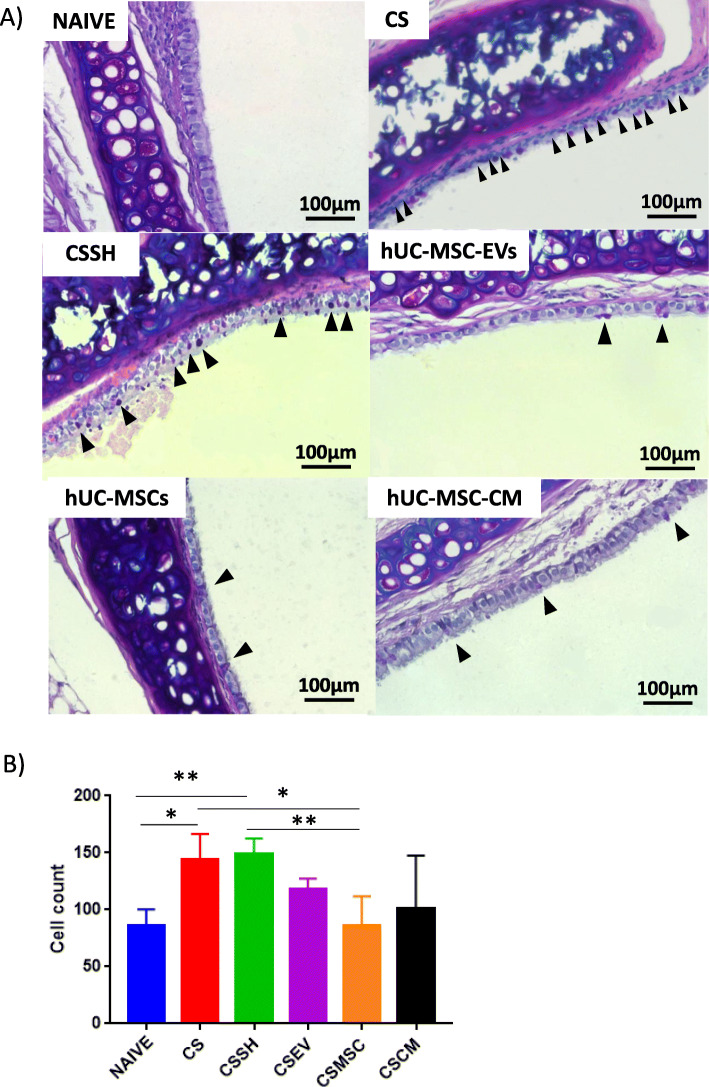
Goblet cell counts for the assessment of mucus overproduction. Quantitative histological staining (**a**) and analysis (**b**) of goblet cells within the bronchi. A significant increase in goblet cells was observed after 12 weeks of CS exposure with no reduction following 2 weeks of self-healing (CSSH). Treatment of the CS groups with MSCs significantly reduced the number of goblet cells and no significant reduction in response to UCMSC-EVs and UCMSC-CM (*p < 0.05, **p < 0.001)

To assess mucus overproduction, the semi-quantitative histological analysis was conducted to count the incidence of goblet cells between the groups (Fig. 7). Histological sections of the bronchi were stained with AB-PAS where the cell nucleus was stained blue, while goblet cells stained magenta (Fig. 7a). Statistical analysis shows that treatment of the CS groups with MSCs had significantly reduced the number of goblet cells (*p* < 0.05) as compared to CS and self-healing (CSSH), and far better than the groups that received treatments with hUCMSC-EVs and hUCMSC-CM (Fig. 7b).

### hUC-MSC-EVs decreased emphysema

**Fig. 8:**
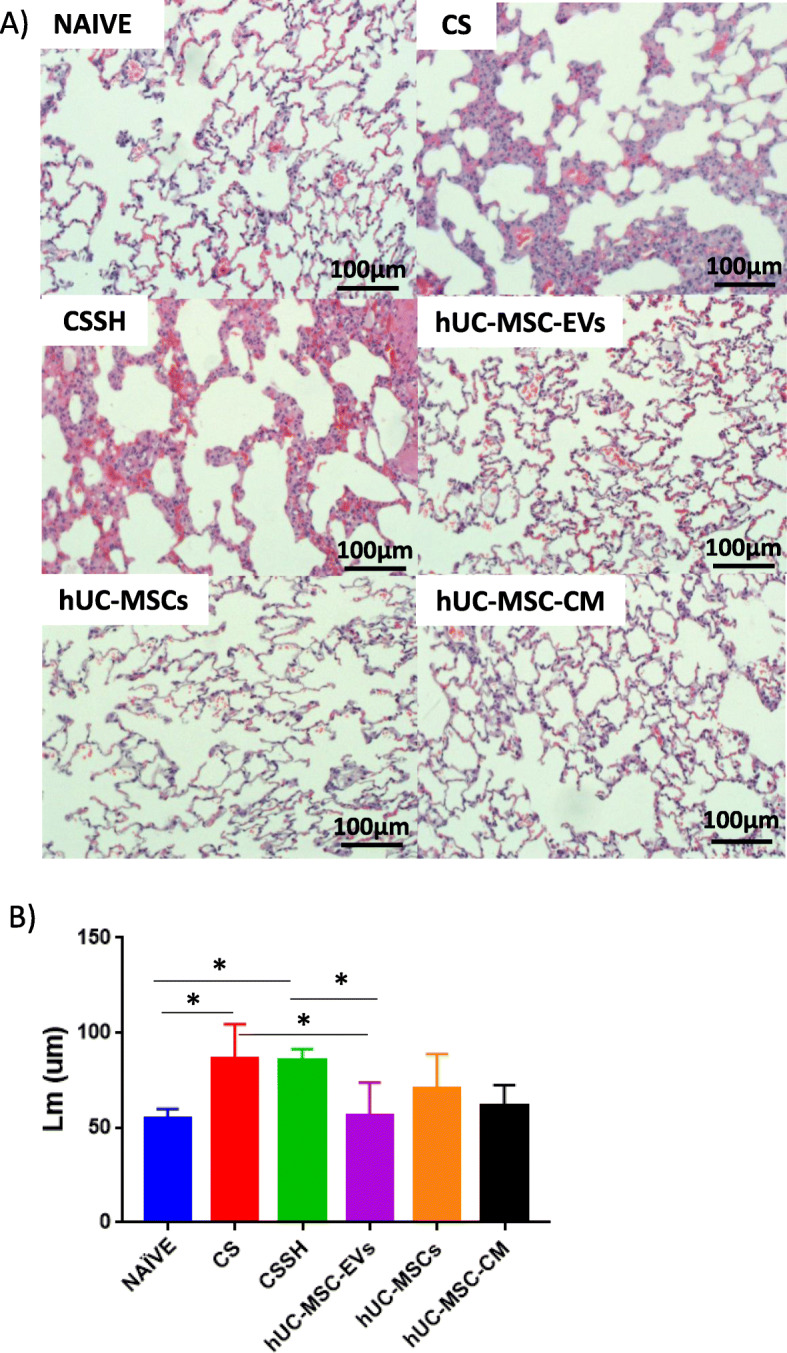
Mean linear intercept of the cigarette smoke-exposed group. Identification of the findings in the figures shown **a** representative histological sections stained with H&E staining for each group. **b** Semi-quantitative analysis of the mean linear intercept of CS-induced emphysema in rat lung. CS increased the mean linear intercept, and 2 weeks of self-healing failed to alleviate the alveolar obstruction. Meanwhile, treatment with hUC-MSC-EVs significantly reduced the mean linear intercept. hUC-MSCs and hUC-MSC-CM did not observe a significant reduction. *p < 0.05

To study the effect of CS and treatment intervention on emphysema, the mean linear intercept of the alveolar pores were measured on H&E histological slides (Fig. 8a). Quantitative analysis showed that 12 weeks of CS exposure caused alveolar destruction with a significant increase in the mean linear intercept of the alveolar pores (Fig. 8b), while 2 weeks of self-healing failed to mitigate these effects (CSSH). However, a significant (*p < 0.05) reduction in the mean linear intercept of the alveolar pores and restoration of tissue was observed following treatment with hUC-MSC-EVs. Meanwhile, a non-significant reduction was observed in hUC-MSCs and hUC-MSC-CM.

### hUC-MSC-EVs decreased the levels of p65 in lung tissue

**Fig. 9:**
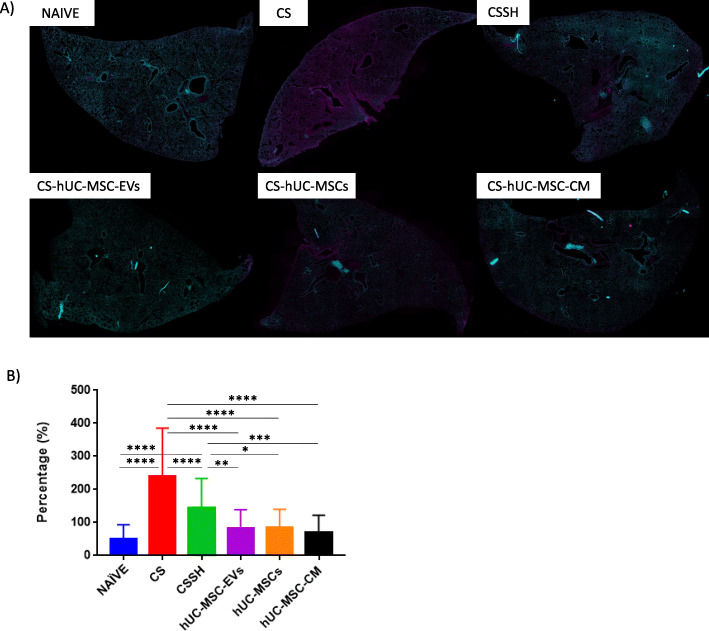
Immunofluorescence staining of CS-exposed lung. Immunofluorescence staining was conducted to study the expression of **a** p65 (purple) and DAPI (turquoise) in the lung. **b** Percentage of p65 expression in all groups. p65 expression was increased when exposed to CS, and smoking cessation for 2 weeks significantly reduced the expression of p65. The expression of p65 was further reduced when treated with hUC-MSCs, hUC-MSC-EVs, and hUC-MSC-CM. *p < 0.05, **p < 0.01, ****p < 0.0001

p65 is a subunit of the prototypic, pro-inflammatory transcription factor NF-κB. Translocation of p65 to the nucleus of a cell is indicative of the cell pro-inflammatory response. To study the translocation of p65 into the nuclei of cells, IHC stained and imaged lung tissue sections were quantified following CS and treatment intervention. A significant increase in the percentage of p65-positive cells was observed in the CS group. Following 2 weeks of self-healing, a significant reduction of p65 was observed. Treatment with hUC-MSC-EVs, hUC-MSCs, and hUC-MSC-CM further reduced the p65 expression in the CS-exposed lung (Fig. 9).

### CS, hUC-MSC-EVs, and hUC-MSCs alter the gene expression

**Fig. 10:**
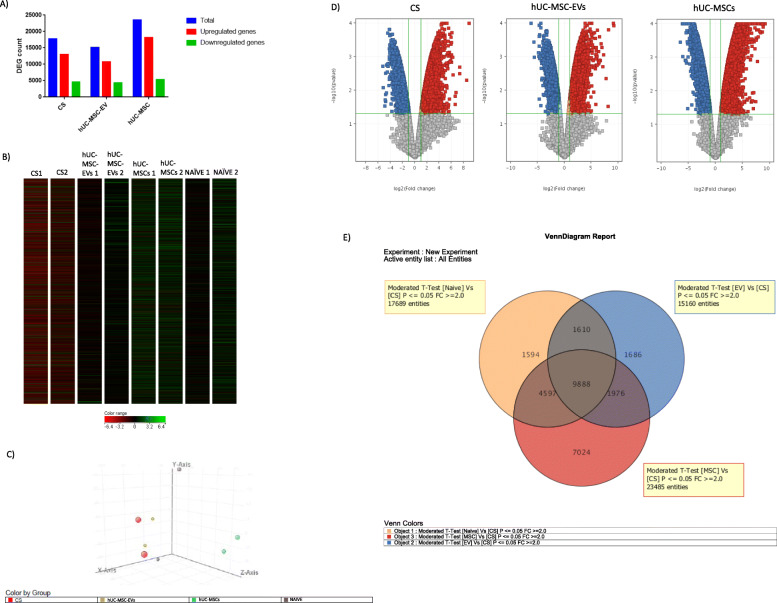
Microarray analysis of significantly regulated genes in the CS-exposed lung. **a** The bar chart represents the total number of DEG, downregulated and upregulated DEG with *p* < 0.05 and FC > 2.0, which are considered significantly regulated. **b** Heat map shows different DEG regulation in the CS group as compared to the naive, hUC-MSC-EVs, and hUC-MSCs groups. **c** PCA plot shows a cluster of samples (*n* = 2) in the naive, CS, hUC-MSC-EVs, and hUC-MSCs groups. **d** Volcano plots of differentially expressed genes obtained from the microarray analysis. The red dots represent upregulated DEG, while blue dots represent downregulated DEG. *p* value generated using *t* test. **e** Venn diagram shows overlapping DEG among CS, hUC-MSC-EVs, and hUC-MSCs. A total of 9888 DEG were overlapped in 3 groups, 1610 DEG were overlapped between CS and hUC-MSC-EVs, 4597 DEG were overlapped between CS and hUC-MSCs, and 1976 DEG were overlapped between hUC-MSC-EVs and hUC-MSCs

Our microarray analysis aimed to determine the pathways and the differential gene expressions altered in the CS-induced lung inflammation and the treatment group (hUC-MSC-EVs and hUC-MSCs). Differentially expressed genes (DEG) which has been upregulated or downregulated more than the two fold difference (*p* < 0.05) are considered significant for further investigation to understand the biological, cellular, and molecular functions. CS exposure was shown to lead to a total of 17,689 DEG, while treatment with hUC-MSC-EVs and hUC-MSCs is shown to have led to 15,160 and 23,485 DEG, respectively (Fig. 10a). The heat map shows the different regulation of DEG from the CS group as compared to the naive, hUC-MSC-EVs, and hUC-MSCs groups (Fig. 10b). The PCA plot shows a cluster of samples (*n* = 2) in the CS, hUC-MSC-EVs, and hUC-MSCs groups, but high variation was observed in the naive group (Fig. 10c). The volcano plot shows DEG in the CS, hUC-MSC-EVs, and hUC-MSCs groups (Fig. 10d). The Venn diagram shows the overlapping DEG among CS, hUC-MSC-EVs, and hUC-MSCs (Fig. 10e). A total of 9888 DEG were overlapped in 3 groups, 1610 DEG were overlapped between CS and hUC-MSC-EVs, 4597 DEG were overlapped between CS and hUC-MSCs, and 1976 DEG were overlapped between hUC-MSC-EVs and hUC-MSCs.

### Gene Ontology analysis

GO slim analysis of the biological process was performed on the DEG results presented in the tables below. The enriched GO terms were identified in the CS (Suppl 1 Table A), hUC-MSCs (Suppl 1 Table B), and hUC-MSC-EVs (Suppl 1 Table C) groups. The GO terms for the CS group are related to the regulation of the cellular process, regulation of catalytic activity, regulation of signalling, and regulation of the metabolic process, while GO terms for hUC-MSC-EVs are related to the regulation of catalytic activity, movement of a cell or subcellular component, regulation of signalling, regulation of cell communication, cellular protein modification process, and regulation of RNA metabolic process. GO terms for hUC-MSCs are related to chemical synaptic transmission, sensory perception of the chemical stimulus, G-protein-coupled receptor signalling pathway, regulation of signalling, regulation of cell communication, ion transport, regulation of biological quality, developmental process, and ribosome biogenesis.

### Pathway analysis

The selection of the regulated pathways related to COPD was determined based on the significant value of *p* < 0.05. Thirty-eight pathways were significantly regulated in response to CS, whereas, following hUC-MSC-EVs treatment, 58 pathways were significantly regulated, and only 17 pathways were significantly regulated following treatment with hUC-MSCs. The notable pathways which were highly regulated in the CS and hUC-MSC-EVs groups include the TGF-β receptor signalling pathway, IL-4 signalling pathway, and TNF-alpha NF-kB signalling pathway. Meanwhile, pathways which were highly regulated in response to the hUC-MSCs group include the TNF-alpha NF-kB signalling pathway, senescence and autophagy pathway, and IL-9 pathways. All the significantly regulated pathways are shown in Suppl 2.

### Gene expression

We look for the highest frequency of genes that are regulated in the pathways in the injury (CS) and treatment (hUC-MSCs and hUC-MSC-EVs) groups. Table 3 shows 10 genes with the highest frequency in CS. NFKB1 and Mapk1 are expressed in 11 and 12 pathways, respectively, followed by Jun and Map 2k1, which are regulated in 10 pathways. Table 4 shows 8 genes with the highest frequency in the hUC-MSC-EVs group. Akt1 is expressed in 22 pathways; meanwhile, Mapk1, NFKB1, and Map 2k1 are regulated in 18 and 15 pathways respectively. Table 5 shows 8 genes with the highest frequency in the UCMSCs group. Akt1 and Mapk1 are regulated in 6 pathways, while Map 2k1 and TGFB1 are regulated in 5 and 4 pathways, respectively.

**Table 3:** Genes with the highest frequency in the CS group

Genes	*p* value	FC	Frequency
Nfkb1	0.0015	6.0905	11
Mapk1 (ERK2)	0.002193779	7.168577	12
Jun	0.041863125	− 3.24888	11
Map 2k1 (MEK1)	0.0097	3.2654	10
Mapk9	0.0026	− 4.401	8
Crebbp (CBP)	0.0089	− 4.07	9
Prkcz	0.0098	3.6678	8
p65	0.0017	6.3732	8
Grb2	0.0235	− 2.237	7
Src	0.0115	− 2.948	7

**Table 4:** Genes with the highest frequency in the hUC-MSC-EVs group

Gene	*p* value	FC	Frequency
Akt1	0.00172	− 4.53957	22
Nfkb1	0.003537	− 4.44685	15
Map 2k1	0.003896	− 3.49505	15
Pik3r1	0.002295	5.379887	14
Mapk1	0.010161	− 3.689022	18
Grb2	0.003507	− 3.40208	12
Prkcz	0.006365	− 3.68258	10
p65	0.001438	− 5.04044	10

**Table 5:** Genes with the highest frequency in the hUC-MSCs group

Gene	*p* value	FC	Frequency
Tgfb1	0.024022	− 5.81517	4
Akt1	0.004107	− 6.88884	6
Mapk1	0.001274641	− 8.08983	4
Pik3r1	0.001114	9.6399	4
Map 2k3	0.002436	− 4.57343	4
Mapk3	0.01819	2.465821	4
Mapk8	0.006342	− 3.81553	4
Map 2k1	3.85E-04	− 9.33631	5

## Discussion

Our study aimed to determine the effects of hUC-MSC-EVs in comparison with hUC-MSCs for the treatment of COPD. The therapeutic potential of MSCs and MSC-derived secreted factors have been widely demonstrated in various diseases, including rheumatoid arthritis, asthma, and Crohn’s disease [33, 64, 77]. In COPD, MSCs’ capabilities to mitigate inflammation have been tested in the preclinical and clinical setting around the world [8, 56, 83]. However, little is known about the effect of extracellular vesicles isolated from MSCs for the treatment of inflammation in COPD. hUC-MSCs used in this study were positive for CD73, CD90, CD105, and CD166 and negative for CD34, CD45, CD31, and HLA DR DP DQ as previously described by [85].

Meanwhile, the differentiation analysis showed the ability of hUC-MSCs to differentiate into adipocyte, osteocyte, and chondrocyte. hUC-MSC-EVs isolated from hUC-MSCs showed a rounded morphology with an average of 153 nm in diameter, and protein analysis showed a positive marker for CD63 exosomal marker. Following 12 weeks of CS exposure, the evidence of accumulation of inflammatory cell infiltrated in peribronchial and perivascular tissues as well as the parenchyma, goblet cell hyperplasia, expression of p65, and the development of emphysema, was consistent to that of previously published studies [62, 94] indicating the development of COPD by CS inhalation. Two weeks of self-healing has significantly reduced the expression of p65 but did not reduce the inflammation and remodelling the destruction of alveolar in the lung. The treatment of hUC-MSC-EVs, hUC-MSCs, and hUC-MSC-CM significantly reversed the effect of sidestream CS on lung inflammation, expression of p65, and emphysema. Our study on microarray also revealed that CS significantly regulated the pathways related to COPD and upregulated genes related to inflammation including NFKB1, p65, and protein kinase Cζ (PRKCZ), while treatment with hUC-MSC-EVs and hUC-MSCs were observed to reverse these CS-induced gene expression effects.

Cigarette smoke is the leading risk factor of COPD, with over 80% of all COPD cases attributed to cigarette smoking. Therefore, cigarette smoke is widely employed by the researchers to develop the in vivo COPD model over other inducers such as biomass fuel, lipopolysaccharide, and elastase [2, 10, 31, 39]. For the establishment of COPD model in animals, the cigarette smoke was exposed to the animals for a 6-month period in order to exhibit the severe injury in the lung [42, 48]. However, there are studies which employed a 12-week cigarette smoke exposure demonstrated characteristic of COPD including inflammation, airway remodelling, fibrosis, goblet cell hyperplasia, and emphysema [35, 40]. This method is more feasible for in vivo study as compared to the 6-month period, which is time-consuming. Our study is in agreement with the previous studies that showed 12 weeks of cigarette smoke exposure is sufficient to induce characteristics similar to COPD in SD rats. Importantly, our method of CS exposure for 2 times/day, 7 days/week for 12 weeks exposure, induced the emphysema in the rat lung, a characteristic of the chronic model of COPD [51]. It should be noted that animal models do not fully mimic human condition, regardless of the types of animal used. The duration of cigarette smoke exposure and the severity of the injury are only equivalent to the Global Initiative for Obstructive Lung Disease (GOLD) stage I or II diseases (Fricker et al. [28]).

COPD is characterized by airway and parenchymal inflammation that leads to mucus overproduction and emphysema, although these characteristics may not be present in all patients, as the emphysematous lung only occurs in 20% of all COPD patients [1, 14]. Nevertheless, in the animal model, the presence of emphysema is one of the important characteristics to confirm the development of COPD [20]. On the other hand, mucus overproduction is considered challenging to reproduce in the rat model due to the low number of goblet cells in the bronchi [14]. Our study using CS exposure for 12 weeks in SD rats successfully developed characteristic of COPD as we can observe the increased influx of immune cells indicating the development of inflammation in the lung, increased goblet cell count which shows increase mucus production, and increased mean linear intercept which shows the development of emphysema.

Airway inflammation begins with the disruption of the airway and vascular function, allowing infiltration of immune cells in the lung [67, 70]. In the acute phase of CS exposure that lasts until the second week, increased neutrophils were observed. After the second week, macrophage begins to increase, and neutrophils start to decrease but not fully resolve, indicating that chronic inflammation began to develop [78]. In our study, the increase in neutrophil, eosinophil, lymphocyte, and macrophage counts was observed; however, neutrophils and macrophages are the predominant immune cells infiltrating the lung. Our results also showed that immune cell accumulation was observed more prominently in the alveolar area rather than the peribronchial and perivascular areas, which destroy the alveolar wall leading to the emphysematous lung.

The accumulation of immune cells in the alveolar walls are prerequisite for the development of emphysema. Neutrophil elastase (NE) was reported to induce the epithelial apoptosis and emphysema; meanwhile, excessive MMP-9 released by macrophage can result in permanent alveolar destruction [6, 41]. Shapiro et al. [73] demonstrated that crosstalk between these two cells is crucial in the development of emphysema. The presence of neutrophils is essential as neutrophils release NE that is required to recruit more neutrophils and monocytes into the lung. The study was also reported that mice deficient of NE (NE^−^/^−^) had shown significantly protected from the development of emphysema. Shapiro and colleagues further proved that the synergistic effects of neutrophil and macrophage are required to enhance the potency of both cells. The absence of NE causes the tissue inhibitors of metalloproteinases (TIMPs) to inhibit the action of macrophage elastase. Likewise, the absence of macrophage elastase caused an increase in α-1 anti-trypsin, a major inhibitor of NE. Thus, the presence of both neutrophils and macrophages is an important factor in the development of emphysema [73].

CS exposure also causes mucus overproduction, although the symptoms may not be present in all COPD patients [11]. The mechanism by which CS induced the overproduction of mucus occurs through activation of TNF-α converting enzyme (TACE) which cleaved pro-TNF-α to release TNF-α that activates epidermal growth factor receptor (EGFR) which results in mucin production [71]. The accumulation of neutrophils in the lung during CS exposure may also exacerbate the mucus overproduction as neutrophils are also in part responsible for the impaired mucociliary clearance, increased goblet cell count, and excessive mucus production. NE released by neutrophils increased the expression of MUC5AC by enhancing the mRNA stability via reactive oxygen species mechanism [5, 25]. Besides, activation of TNF-α and subsequent activation epidermal growth factor pathway can also stimulate NE to induce the expression of MUC5AC [49].

MSCs have been actively investigated as a potential therapy for COPD. Clinical studies measuring C-reactive protein in COPD patient revealed the benefit of MSC administration in mitigating the inflammation [37]. In the animal model, MSCs alleviate the inflammation by reducing the alveolar macrophage, while at the same time promoting the expression of the anti-inflammatory cytokine, IL-10, in macrophages [35]. MSCs also reduced the neutrophil infiltration regardless of the route of administration [4]. This therapeutic effects of MSCs are governed by the release of paracrine factors, including growth factor, cytokine, and EVs rather than cell-to-cell contact [27]. Recently, research begins to unravel the therapeutic effects of MSC-derived EVs and better understand the mechanism behind this ability. Several studies have shown anti-inflammatory effects of MSC-derived EVs in mitigating the inflammation similar to MSCs. Maremanda et al. [59] study the effect of MSC, MSC-exosomes, and combination of MSC + MSC-exosomes in acute CS exposure in mouse model. The group measured the total cell count and differential cell count in BAL fluid. The treatment of MSC, MSC-exosomes, and combination of MSC + MSC-exosomes decreased the total cell count, macrophages, neutrophils, and CD4^+^ T cell count. However, the group did not measure the accumulation of immune cells in the peribronchial and parenchyma areas [59]. Apart from CS-induced inflammation, MSC-exosomes also have been shown to modulate the differentiation, activation, and proliferation of T cells in vitro [9]. Reduced number of eosinophils, lymphocytes, and airway remodelling were observed in the animal model of asthma when treated with adipose tissue MSC-EVs [18]. In the rat model of hepatic ischemia-reperfusion injury, hUC-MSC-EVs inhibited the activity of the neutrophils by attenuation of respiratory burst and oxidative stress, thus reducing the apoptosis of hepatocytes [90]. Also, MSC-EVs attenuated the pro-inflammatory cytokines such as IL-17, TNF-α, RANTES, MIP1α, MCP-1, CXCL1, and HMGB1 while enhancing the production of IL-10, PGE2, and KGF [79]. In agreement with the previous studies, our study demonstrated that hUC-MSC-EVs possess anti-inflammatory similar to its cell counterpart, hUC-MSCs. The treatment with hUC-MSC-EVs significantly reduced immune cell accumulation in the lung, especially neutrophil accumulation, reduced emphysema, reduced protein expression of p65, and downregulated DEG related to COPD.

To date, there are no treatment options available to regenerate the lung damage in emphysema. However, stem cell-based therapy demonstrates a promising regenerative capability to restore the function of the damaged lung. MSCs and MSC-CM are shown to restore the lung function by mitigating the apoptosis in the emphysematous lung [42]. This anti-apoptosis effect is in part mediated by vascular endothelial growth factor (VEGF) and VEGF receptor [36]. Besides, MSCs reduced the expression of cyclooxygenase-2 in alveolar macrophage, thereby mitigating the emphysema in a rat model of COPD [35].

On the other hand, relatively few studies were conducted to decipher the effects of MSC-derived EVs in the emphysematous lung. The study by Kim et al. [47] compared the regenerative effects of nanovesicles generated from adipose stem cells (ASC) and ASC-derived exosomes in the elastase-induced emphysematous lung. The result showed that nanovesicles significantly reduced the emphysema via its cargo content, FGF2, while no significant reduction of emphysema was observed in ASC-derived exosome [47]. In a study examining the effect of MSC-exosome on bronchopulmonary dysplasia, a chronic lung disease in the preterm infant, characterized by restricted lung growth, subdued alveolar and blood vessel development and impaired pulmonary function; MSC-exosomes are shown to reduce the mean linear intercept, while increasing the lung alveolarization, through alteration of macrophage pro-inflammatory M1 phenotype into anti-inflammatory M2 phenotype [84]. Our result provides the evidence of hUC-MSC-EVs ability to reduce emphysema in CS-induced COPD in a rat model. Considering the importance of neutrophil and macrophage accumulation in the pathogenesis of emphysematous lung, a significant reduction in the accumulation of neutrophils when treated with hUC-MSC-EVs and hUC-MSCs in our study in part might explain the reduction of emphysema. A decrease in macrophage accumulation was also observed when treated with hUC-MSC-EVs, hUC-MSCs, and hUC-MSC-CM, although the reduction was not significantly different from the injury group. Recent studies also reported that MSC-derived microvesicles reduced the influx of neutrophils through the effects of KGF [97]. However, macrophages are shown to play an essential role in MSC anti-inflammatory effects by changing from M1 to M2 phenotype which produces IL-10 that involve in the reduction of inflammation when treated with MSCs and MSC-EVs [24, 35, 75]. Although the accumulation of macrophages is prerequisite for emphysema, however, in allergic asthma, depletion of alveolar macrophage reversed the immunosuppressive effect of MSCs in which the production of IL-10 was dependent on the presence of alveolar macrophage [60]. The macrophages’ role might explain why macrophages in our study did not significantly reduce as it aids in MSC anti-inflammatory response.

To date, relatively few studies are examining the effect of MSCs in reducing the mucus overproduction. Although there are reports stating that mucus could be mitigated with the administration of MSCs, in-depth analysis of the mechanism involves remaining unknown [53, 61]. Besides, there is no report on the ability of MSC-EVs to reduce mucus overproduction. Our study showed a significant reduction of goblet cell count in hUC-MSCs. Reduction of goblet cells can be observed in hUC-MSC-EVs and hUC-MSC-CM; however, the reduction was not significant. The extracellular environment can alter the MSCs’ fate and the paracrine factors released by the MSCs [80]. Thus, the hUC-MSCs transplanted into the lung will be influenced by the lung microenvironment, and the paracrine factors that are being released by the transplanted MSCs will be different from the hUC-MSC-EVs and hUC-MSC-CM collected from the hUC-MSCs grown in the flask, thereby will affect the lung differently. This effect can be observed in the various pathways that hUC-MSCs and hUC-MSC-EVs regulated in our study. We also speculate that hUC-MSC-EVs and hUC-MSC-CM can affect the lung tissues faster than hUC-MSCs, as hUC-MSCs will also need to establish cell-to-cell contact, and the lung microenvironment will also have to communicate with hUC-MSCs, in order for hUC-MSCs to produce an effect. Meanwhile, MSC-EVs and paracrine factors in MSC-CM can readily be taken up by the cells in the lung due to its small size [19]. Hence, we observed more reduction of goblet cell count in the MSC-EVs and MSC-CM treatment groups as compared to MSCs alone.

Our microarray analysis aimed to determine the pathways associated with COPD and gene expression profile in our COPD model. We also seek to understand how the treatment with hUC-MSC-EVs and hUC-MSCs can change the gene expression profile and pathways in COPD model. Our DEG analysis of microarray data revealed the importance of p50, p65, and PRKCZ in our animal model. Twelve weeks of CS exposure significantly upregulated p50, p65, and PRKCZ, and the treatment with hUC-MSC-EVs significantly downregulated the expression of these genes. Immunohistochemistry staining on p65 confirms the significant upregulation of p65 protein in the CS group and significant downregulation of p65 when treated with hUC-MSC-EVs, hUC-MSCs, and hUC-MSC-CM. Our study was also revealed that p50, p65, and PRKCZ were involved in many pathway regulations that include the TNF-α NF-κβ signalling pathway, IL-2 signalling pathway, oxidative stress, oestrogen signalling pathway, and IL-4 signalling pathway.

The expression of PRKCZ and NF-κβ play a vital role in inflammation and thus the pathogenesis of COPD. PRKCZ is upstream of NF-κβ, phosphorylating p65 at serine 311 to promote the acetylation of Lysine 310, thus activating the κβ transcription [22]. Mice deficient of PRKCZ was found to reduce myeloperoxidase and influx of neutrophils, and reduced pro-inflammatory cytokines such as IL-13, IL-17, IL-18, IL-1β, TNF-α, MCP-1, MIP-2, and IFN-γ, while the use of PRKCZ inhibitors blocked the activation of NF-κβ by TNF-α, thus reducing the pro-inflammatory IL-8 expression [7, 89]. Meanwhile, NF-κβ was composed of five members, NF-κβ1 (p50), NF-κβ2 (p52), RelA (p65), RelB, and c-Rel, that regulate a multitude of genes involved in inflammatory responses [55]. Among all heterodimers of NF-κβ, p50/p65 heterodimer represents the most abundant NF-κβ activated by the canonical pathway [32].

Cigarette smoke activates NF-κβ within 1 h of exposure to the lung thus causing inflammatory reactions which increase white blood cell count, lymphocyte count, and granulocyte count [15, 26]. Data from the pre-clinical study showed 4 weeks of CS exposure significantly increased p65 and Iκβα in mouse lung as compared to the control group [92]. NF-κβ is also required by IL-1β and IL-17A to induce the expression of MUC5B in bronchial epithelial cells that cause goblet hyperplasia in COPD [30]. Besides, various studies demonstrated the upregulation of p65 and p50 expression in COPD patients [12, 21, 81, 96]. Microarray study conducted by Yang et al. [87] revealed the vital role of p50 in regulating many pathways of COPD including Toll-like receptor signalling pathway, cytokine-cytokine receptor interactions, chemokine signalling pathway, and apoptosis [87].

Our study revealed the downregulation of PRKCZ, p65, and p50 expression when treated with hUC-MSC-EVs. p50 regulated 18 pathways in the hUC-MSC-EVs group, while PRKCZ and p65 regulated 10 pathways suggesting the vital role of the NF-κβ pathway in hUC-MSC-EVs therapeutic effects in our model. Downregulation NF-κβ subunit by hUC-MSC-EVs can affect multiple pathways in our model, thus reducing the inflammation. MSC-EVs have been shown to decrease the expression of NF-κβ in an in vitro model of cystic fibrosis and experimental colitis [88, 98]. MSC-exosomes also interfered with TLR-4 signalling of BV2-microglia, which prevented the degradation of NFκβ inhibitor, Iκβα, and phosphorylation of MAPK family protein in response to LPS stimulation [82]. However, much is still unknown about how MSC-EVs regulate the NF-κβ pathway. In our study, we did not elucidate the cargo content of hUC-MSC-EVs that is responsible for the anti-inflammatory effects on CS-induced lung inflammation. Nevertheless, the study demonstrated that micro-RNA content of MSC-derived exosome could reduce p50 NF-κβ pathway in macrophage, thus preventing the Toll-like receptor-induced macrophage activation [65]. In addition, CCR2 in MSC-derived exosomes abolished the ability of CCL2 to induce p65 phosphorylation in macrophages [74]. Meanwhile, knockdown of GPX-1 in human MSCs,reverses the effect of MSC-derived exosomes in reducing the phosphorylation of p65 [86]. These results proved that multiple cargo contents of MSC-EVs play a vital role in mediating the inflammation.

## Conclusion

Our study had successfully isolated the hUC-MSC-EVs from hUC-MSCs. Twelve weeks of CS exposure induced the inflammation and increased goblet cell count and emphysema in the rat model. The treatment with hUC-MSC-EVs, hUC-MSCs, and hUC-MSC-CM decreased the inflammation in the lung and decreased the goblet cells, and destruction of the lung in a rat model of COPD similar to hUC-MSCs. hUC-MSC-EVs reduced the inflammation in part by the expression of PRKCZ, and NF-κβ subunits p65 and p50, which regulates many genes responsible for innate and adaptive immune response. Confirmation study using immunofluorescence on p65 showed a similar result as microarray analysis of DEG. Taken together, there are still limited data demonstrating the regenerative and the anti-inflammatory effects of MSC-EVs to mitigate the inflammation in COPD. More studies should be conducted to decipher the anti-inflammatory effects of MSC-EVs as a whole, as well as exosomes, and microvesicles as different particle might exhibit different therapeutic effects. Determination of cargo content of MSCEVs responsible for the anti-inflammatory effects and the mechanism of action of the cargo content of MSC-EVs can provide a clear with the ways toward the goal of using hUC-MSCs as a new treatment for COPD.

## Supplementary Information

**Additional file 1: Suppl 1.** GO Slim analysis of the biological process in CS (A), hUC-MSCs group (B) and hUC-MSC-EVs (C) groups.

**Additional file 2: Suppl 2.** Significantly regulated pathway in CS-induced inflammation, hUC-MSC-EVs and hUC-MSCs. Thirty-eight pathways are significantly regulated in CS. 58 pathways are significantly regulated in hUC-MSC-EVs. Only 17 pathways are significantly regulated in hUC-MSCs. Pathway with *p* < 0.05 is considered as significantly regulated.

## Data Availability

Not applicable

## Abbreviations

COPD: Chronic obstructive pulmonary disease
EVs: Extracellular vesicles
hUC-MSCs: Human umbilical cord mesenchymal stem cell
AB-PAS: Alcian blue-periodic acid-Schiff
ANOVA: One-way analysis of variance
SD: Standard deviation
CS: Cigarette smoke
MSCs: Mesenchymal stem cells
BM: Bone marrow
UC: Umbilical cord
AT: Adipose tissue
MVB: Multivesicular bodies
mRNA: Messenger ribonucleic acid
miRNA: Microribonucleic acid
IL: Interleukin
iNOS: Inducible nitric oxide synthase
MDA: Malondialdehyde
MPO: Myeloperoxidase
SOD: Superoxide dismutase
GSH: Glutathione
FBS: Foetal bovine serum
DMEM: Dulbecco’s modified Eagle’s medium
ISCT: International Society for Cellular Therapy
CD: Cluster of differentiation
HLA: Human leukocyte antigen
NTA: Nanoparticle tracking analysis
PVDF: Polyvinylidene difluoride
H&E: Haematoxylin and eosin
Lm: Mean linear intercept
WBC: White blood count
DEG: Differentially expressed genes
NF-kB: Nuclear factor kB
GO: Gene Ontology
TGF-β: Transforming growth factor-β
TNF-alpha: Tumor necrosis factor-alpha
Map 2k1: Dual specificity mitogen-activated protein kinase kinase 1
Akt1: RAC-alpha serine/threonine-protein kinase
MAPK: Mitogen-activated protein kinase
Crebbp: Creb-binding protein
Prkcz: Protein kinase C zeta
Grb2: Growth factor receptor-bound protein 2
Src: Proto-oncogene tyrosine-protein kinase
Pik3r1: Phosphatidylinositol 3-kinase regulatory subunit alpha
Map 2k3: Mitogen-activated protein kinase kinase 3
