# Supplementary material for: Human umbilical cord mesenchymal stem cell-derived extracellular vesicles ameliorate airway inflammation in a rat model of chronic obstructive pulmonary disease (COPD)
Source: Stem Cell Res Ther. 2021 Jan 12;12:54. doi: 10.1186/s13287-020-02088-6 (PMC7805108; doi:10.1186/s13287-020-02088-6)
Supplement: Supplementary file 1 — Additional file 1: Suppl 1. GO Slim analysis of the biological process in CS (A), hUC-MSCs group (B) and hUC-MSC-EVs (C) groups. [file 13287_2020_2088_MOESM1_ESM.docx]

**Suppl 1: GO Slim analysis of the biological process in CS (A), hUC-MSCs group (B) and hUC-MSC-EVs (C) groups.**

**Table A**

| **Category Name** | **Accession** | **Fold enrichment** | **p-value** |
| --- | --- | --- | --- |
| **Positive regulation of organelle organization** | GO:0010638 | 1.62 | 0.00107 |
| [**Positive regulation of cellular component organization**](http://pantherdb.org/panther/category.do?categoryAcc=GO:0051130) | GO:0051130 | 1.49 | 0.000449 |
| [**Regulation**](http://pantherdb.org/panther/category.do?categoryAcc=GO:0050794) **of cellular process** | GO:0050794 | 1.07 | 0.00131 |
| **Regulation of biological process** | GO:0050789 | 1.07 | 0.000552 |
| **Biological regulation** | GO:0065007 | 1.07 | 0.000258 |
| **Glycerolipid metabolic process** | GO:0046486 | 1.48 | 0.00125 |
| **Cellular metabolic process** | GO:0044237 | 1.07 | 0.000548 |
| **Cellular process** | GO:0009987 | 1.05 | 0.000423 |
| **Regulation of catalytic activity** | GO:0050790 | 1.21 | 0.00122 |
| **Regulation of molecular function** | GO:0065009 | 1.21 | 0.000509 |
| **Regulation of signal transduction** | GO:0009966 | 1.20 | 0.00115 |
| **Regulation of cell communication** | GO:0010646 | 1.21 | 0.000365 |
| **Regulation of signaling** | GO:0023051 | 1.21 | 0.000354 |
| **Regulation of primary metabolic process** | GO:0080090 | 1.12 | 0.000529 |
| **Regulation of metabolic process** | GO:0019222 | 1.12 | 0.000561 |
| **Regulation of nitrogen compound metabolic process** | GO:0051171 | 1.12 | 0.000640 |
| **Regulation of cellular metabolic process** | GO:0031323 | 1.12 | 0.000642 |
| **Regulation of macromolecule metabolic process** | GO:0060255 | 1.12 | 0.000778 |

**Table B:**

| **Category Name** | **Accession** | **Fold enrichment** | **p-value** |
| --- | --- | --- | --- |
| **Chemical synaptic transmission** | GO:0007268 | 1.36 | 0.0000312 |
| **Anterograde trans-synaptic signalling** | GO:0098916 | 1.36 | 0.0000312 |
| **Trans-synaptic signaling** | GO:0099537 | 1.35 | 0.0000473 |
| **Synaptic signaling** | GO:0099536 | 1.35 | 0.0000501 |
| **Cell-cell signaling** | GO:0007267 | 1.26 | 0.000132 |
| **Signalling** | GO:0023052 | 1.10 | 0.000104 |
| **Cell communication** | GO:0007154 | 1.10 | 0.000181 |
| **Sensory perception of chemical stimulus** | GO:0007606 | 1.29 | 0.00117 |
| **Sensory perception** | GO:0007600 | 1.28 | 0.000764 |
| **Nervous system process** | GO:0050877 | 1.28 | 0.000111 |
| **System process** | GO:0003008 | 1.26 | 0.000113 |
| **Multicellular organismal process** | GO:0032501 | 1.15 | 0.0000231 |
| **G protein-coupled signaling pathway** | GO:0007186 | 1.26 | 0.000000564 |
| **Signal transduction** | GO:0007165 | 1.08 | 0.00165 |
| **Regulation of cellular process** | GO:0050794 | 1.07 | 0.000121 |
| **Regulation of biological process** | GO:0050789 | 1.07 | 0.0000510 |
| **Biological regulation** | GO:0065007 | 1.08 | 0.00000941 |
| **Cellular response to a stimulus** | GO:0051716 | 1.08 | 0.00141 |
| **Regulation of signaling** | GO:0023051 | 1.17 | 0.000448 |
| **Regulation of cell communication** | GO:0010646 | 1.17 | 0.000519 |
| **Ion transport** | GO:0006811 | 1.17 | 0.00123 |
| **Regulation of biological quality** | GO:0065008 | 1.15 | 0.000382 |
| **Developmental process** | GO:0032502 | 1.12 | 0.00121 |

**Table C:**

| **Catergory Name** | **Accession** | **Fold enrichment** | **p-value** |
| --- | --- | --- | --- |
| **Regulation of catalytic activity** | GO:0050790 | 1.24 | 0.000716 |
| **Regulation of molecular function** | GO:0065009 | 1.26 | 0.0000404 |
| **Biological regulation** | GO:0065007 | 1.08 | 0.000199 |
| **Movement of cell or subcellular component** | GO:0006928 | 1.24 | 0.000907 |
| **Cellular process** | GO:0009987 | 1.05 | 0.000295 |
| **Regulation of signaling** | GO:0023051 | 1.20 | 0.00133 |
| **Regulation of biological process** | GO:0050789 | 1.08 | 0.000352 |
| **Regulation of cell communication** | GO:0010646 | 1.20 | 0.00140 |
| **Cellular protein modification process** | GO:0006464 | 1.17 | 0.000541 |
| **Protein modification process** | GO:0036211 | 1.17 | 0.000541 |
| **Macromolecule modification** | GO:0043412 | 1.17 | 0.000327 |
| **Cellular macromolecule metabolic process** | GO:0044260 | 1.09 | 0.000749 |
| **Cellular metabolic process** | GO:0044237 | 1.09 | 0.0000636 |
| **Regulation of RNA metabolic process** | GO:0051252 | 1.15 | 0.00134 |
| **Regulation of macromolecule metabolic process** | GO:0060255 | 1.14 | 0.000108 |
| **Regulation of metabolic process** | GO:0019222 | 1.15 | 0.0000697 |
| **Regulation of nucleobase-containing compound metabolic process** | GO:0019219 | 1.15 | 0.000826 |
| **Regulation of primary metabolic process** | GO:0080090 | 1.16 | 0.0000409 |
| **Regulation of cellular metabolic process** | GO:0031323 | 1.15 | 0.000119 |
| **Regulation of nitrogen compound metabolic process** | GO:0051171 | 1.15 | 0.0000827 |
