# Supplementary material for: Human umbilical cord mesenchymal stem cell-derived extracellular vesicles ameliorate airway inflammation in a rat model of chronic obstructive pulmonary disease (COPD)
Source: Stem Cell Res Ther. 2021 Jan 12;12:54. doi: 10.1186/s13287-020-02088-6 (PMC7805108; doi:10.1186/s13287-020-02088-6)
Supplement: Supplementary file 2 — Additional file 2: Suppl 2. Significantly regulated pathway in CS-induced inflammation, hUC-MSC-EVs and hUC-MSCs. Thirty-eight pathways are significantly regulated in CS. 58 pathways are significantly regulated in hUC-MSC-EVs. Only 17 pathways are significantly regulated in hUC-MSCs. Pathway with p < 0.05 is considered as significantly regulated. [file 13287_2020_2088_MOESM2_ESM.docx]

**Suppl 2: Significantly regulated pathway in CS-induced inflammation, hUC-MSC-EVs and hUC-MSCs.** Thirty-eight pathways are significantly regulated in CS. 58 pathways are significantly regulated in hUC-MSC-EVs. Only 17 pathways are significantly regulated in hUC-MSCs. Pathway with p<0.05 is considered as significantly regulated.

| **Pathways** | **P-value** | | |
| --- | --- | --- | --- |
|  | **CS** | **hUC-MSCs-EVs** | **hUC-MSCs** |
| **Metapathway biotransformation** | 1.18E-04 | 1.71E-05 | 9.53E-04 |
| **TGF-beta Receptor Signaling Pathway** | 1.41E-04 | 3.64E-05 | 0.022073 |
| **G Protein Signaling Pathways** | 0.001383 | 0.001299 | 0.00519 |
| **IL-4 Signaling Pathway** | 0.001675 | 5.63E-04 |  |
| **Androgen Receptor Signaling Pathway WP68** | 0.002252 | 0.01302 | - |
| **CDKN1A-EGF-CREB** | 0.002428 | 8.84E-06 |  |
| **Myometrial Relaxation and Contraction Pathways** | 0.003123 | 1.84E-05 | - |
| **Glucuronidation** | 0.00359 | 0.03215 | 0.033907 |
| **Estrogen signalling** | 0.003774 | 2.31E-06 |  |
| **Endochondral Ossification** | 0.005134 | 0.003877 | 0.019258 |
| **TNF-alpha NF-kB Signaling Pathway** | 0.005701 | 8.29E-06 |  |
| **Calcium Regulation in the Cardiac Cell** | 0.00619 | 2.91E-06 | 2.38E-04 |
| **Hypothetical Network for Drug Addiction** | 0.006872 | 0.049288 | 0.045782 |
| **IL-9 Signaling Pathway** | 0.007162 | 0.040913 | 0.034966 |
| **Focal Adhesion** | 0.007421 | 1.18E-04 | 0.040451 |
| **Glycolysis and Gluconeogenesis** | 0.013374 | 0.046473 | - |
| **Selenium metabolism Selenoproteins** | 0.013374 | 0.022909 | - |
| **Eukaryotic Transcription Initiation** | 0.014185 | 1.96E-04 | - |
| **mRNA processing** | 0.015973 | 0.002469 | - |
| **IL-2 Signaling Pathway** | 0.016009 | 0.001944 | - |
| **p53 pathway** | 0.016763 | 0.001902 | - |
| **Folic Acid Network** | 0.019512 | 0.003951 | - |
| **Oxidative Stress** | 0.019512 | 0.003951 | - |
| **GPCRs,Class A Rhodopsin-like** | 0.020691 | 0.004283 | 0.005291 |
| **Senescence and Autophagy** | 0.021184 | 7.46E-04 | 0.030636 |
| **ErbB signalling pathway** | 0.024823 | 0.007723 | - |
| **NFE2L2** | 0.036411 | 0.001355 | - |
| **Glutathione metabolism** | 0.036946 | 0.002997 | - |
| **Hedgehog Signaling Pathway** | 0.036946 | 0.002997 | - |
| **Apoptosis Modulation by HSP70** | 0.038583 | 0.037473 | - |
| **Irinotecan Pathway** | 0.040463 | 0.002913 | - |
| **IL-1 Signaling Pathway** | 0.048728 | 0.048158 | - |
| **Selenium Micronutrient Network** | 0.049905 | 0.004441 | - |
| **GPCRs, Class C Metabotropic glutamate, pheromone** | 0.022562 | - | - |
| **Renin-Angiotensin System** | 0.024823 | - | - |
| **D-Glucose-Ins1-Rxra** | 0.033369 | - | - |
| **Triacylglyceride Synthesis** | 0.021175 | - | - |
| **TGF-Beta Signaling Pathway** | 0.020114 | - | - |
| **Cytokines and Inflammatory Response** | - | 0.049463 | 0.035269 |
| **Toll-like receptor signaling pathway** | - | 0.035379 | 0.034536 |
| **Signal Transduction of S1P** | - | 0.023728 | 0.025022 |
| **Monoamine GPCRs** | - | 0.042178 | 0.025745 |
| **Insulin Signaling** | - | 0.003641 | - |
| **PKA-HCG-Glycogen Synthase** | - | 0.004314 | - |
| **Apoptosis** | - | 0.006595 | - |
| **Relationship between glutathione and NADPH** | - | 0.008007 | - |
| **Alpha6-Beta4 Integrin Signaling Pathway** | - | 0.008108 | - |
| **Complement Activation, Classical Pathway** | - | 0.013549 | - |
| **Striated Muscle Contraction** | - | 0.01622 | - |
| **Adipogenesis** | - | 0.017211 | - |
| **PI3K AKT NFKB pathway** | - | 0.01997 | - |
| **Glycogen Metabolism** | - | 0.026467 | - |
| **TCA Cycle** | - | 0.029885 | - |
| **Serotonin and anxiety** | - | 0.030435 | - |
| **Cardiovascular Signaling** | - | 0.031013 | - |
| **Id Signaling Pathway** | - | 0.033264 | - |
| **IL-6 Signaling Pathway** | - | 0.034719 | - |
| **EGFR1 Signaling Pathway** | - | 0.037561 | - |
| **Wnt Signaling Pathway** | - | 0.038123 | - |
| **IL-7 Signaling Pathway** | - | 0.040049 | - |
| **Synthesis and Degradation of Ketone Bodies** | - | 0.041841 | - |
| **p38 MAPK Signaling Pathway** | - | 0.042178 | - |
| **Translation Factors** | - | 0.044475 | - |
| **Keap1-Nrf2** | - | - | 0.008516 |
| **Fatty Acid Omega Oxidation** | - | - | 0.021907 |
